# Effect of submucosal alcohol injection on prolonged rectal prolapse in infants and children

**DOI:** 10.4103/0971-9261.42566

**Published:** 2008

**Authors:** Abi Bahador, Hamid Reza Foroutan, Seyed Mohammad Vahid Hosseini, Sam Zeraatian Nejad Davani

**Affiliations:** Department of Pediatric Surgery, Nemazee Hospital, Shiraz University of Medical Sciences, Shiraz - 71877-33398, Iran

**Keywords:** Ethyl alcohol, rectal prolapse, sclerosing agents

## Abstract

**Aim:**

Our aim in this study is to evaluate the effect of ethanol as a sclerosing agent on subset of pediatric patients with prolonged rectal prolapse.

**Materials and Methods:**

From 1997 to 2003, 165 cases of primary rectal prolapse were treated by submucosal injection of ethyl alcohol (96%) after 8 weeks of conservative therapy. Around 1.5-2 ml of alcohol was linearly injected in three sites (two laterals and one posterior).

**Results:**

Twelve of the 165 cases lost the follow-up and 153 cases were followed from 9 months to 6 years. One hundred and six patients (69.3%) had a duration of prolapse for 3-7 months. Forty patients (26.1%) had prolapse for more than 7 months and seven patients had prolapse for more than 1 year. One hundred and forty-seven out of 153 (96%) patients responded to single injection. Three of the children required a second injection. Three patients with age of more than 13 did not respond to the treatment. Twenty five cases had fecal soilage for few days. No infectious complication and no recurrence were observed.

**Conclusion:**

We concluded that 4-6 ml of ethyl alcohol (96%) is effective for the treatment of rectal prolapse. The duration of rectal prolapse had no deleterious effect on treatment; however, patients with age more than 13 years did not respond to sclerosing agent, probably due to different etiology.

## INTRODUCTION

Rectal prolapse is a benign and self-limited condition that causes considerable anxiety for the child and his family according to severity of the disease.[1] Two types of rectal prolapse have been described. One type is less pronounced and intermittent that responds to conservative treatment after a few weeks. The other type is more pronounced and lasts several weeks or months.[1] Many types of treatments, including injection of sclerosing agents, has been described; however, to the best of our knowledge, alcohol is used as a sclerosing agent only in one report and its effect on prolonged rectal prolapse has not been investigated.[2] Herein, we report our studies on patients suffering from prolonged rectal prolapse, who were treated by submucosal alcohol injection.

## MATERIALS AND METHODS

From October 1997 to March 2003, 165 cases of rectal prolapse were treated in our center by injection of 96% ethyl alcohol. Prolapse with secondary causes (cystic fibrosis, neurological causes, bladder extrophy, imperforate anus, etc.) were excluded. The children underwent conservative treatment for 8 weeks before alcohol injection. All the patients had anorectal manometry before the procedure to exclude the specific causes. Data of age, sex, episodes of injection, complications and recurrence were collected. The procedure was performed as out -patient day-care procedure without bowel preparation. In lithotomy position and under general anesthesia, the left index finger was introduced into the rectum. A spinal needle number 19 or 21 was mounted on to a syringe. The needle was inserted from the mucocutaneous junction and advanced submucosally in full length, while the index finger palpated the needle. During withdrawing the needle 1.5-2 ml of alcohol was injected linearly in three sites (two lateral sites and one posterior site). Patients were discharged with analgesia, but without antibiotics. Follow-up was carried out on day 5, 14, 30 and then every 3 months for 1 year.

## RESULTS

On hundred and sixty-five children were treated for rectal prolapse with the injection of ethyl alcohol. Twelve patients were lost in the follow-up. One hundred and fifty-three cases (108 male, 45 female) had a mean age of 2.2 years (range: 9 months to 5 years). The minimum follow-up was 9 months. Anorectal manometry and sweat chloride test were normal in all the patients. According to the duration of prolapse, the patients were divided into three groups. Group 1 (*n* = 106) had a duration of rectal prolapse for 3-7 months; Group 2 (*n* = 40), 8-12 months and Group 3 (*n* = 7), >1 year i.e., 1-5 years [Table 1].

**Table 1:** Comparison of the cure rate of rectal prolapse with different durations of the disease

Group	1	2	3
	N=106	N=40	N=7
Duration of prolapse (months)	3-7	8-12	>12
Response to single injection	103	38	6
Response to two injections	2	1	-
No response	1	1	1

One hundred and forty-seven patients had good response to single injection (96%). Three patients (2%) required a second injection. Three (2%) patients had no response after the second injection and were treated by other procedures. The age of patients who did not respond was more than 13 years, and the duration of the prolapse was 6, 11 and 14 months. Twenty-five cases (16.4%) developed fecal soilage for a few days. Four patients presented with limping for 2-3 days. No infectious complications occurred and no recurrence was observed after the postoperative follow-up at 1 year.

## DISCUSSION

The sex incidence in our study was 2.4:1 which is different to what reported by Corman *et al*. (1;1) and Chan *et al*. (1.8:1). The mean age of our patients was 2 years and 3 months that is similar to other reports.[2]

According to the severity and need for surgery for rectal prolapse, Quist *et al*.[1] described two types. One type that is less pronounced with response to the conservative treatment within 1-8 weeks. The other one is more pronounced and may continue for several weeks or months; this required surgical intervention. All our patients were treated 8 weeks after the onset of their disease to ensure that the conservative treatment has failed.

Most of our patients were from rural areas and the parents believed that this disease subsided spontaneously. Probably, this belief was the cause of delay in seeking of medical attention in our patients. Many agents have been used as sclerosing agents in the treatment of rectal prolapse. Kay[4] and Dutta[5] used 9-15 ml of 30% saline pararectally with the success rates of 78.4% and 83.4%, after the first injection and 94% and 96.7% after the second injection.

Wyllie[6] used 8-10 ml of 5% phenol in almond oil with the success rates of 91% after the first injection and 100% after the second injection. Chan *et al*.[3] used 1 ml/kg of 50% dextrose in water and other agents with the success rates of 64% after the first injection and 84% after the second injection. The failure percentage (16%) after the second injection was probably due to the involvement of secondary rectal prolapse.

We found only one report in which alcohol was used as the sclerosing agent. Melyshev *et al*.[7,8] used (upto 35 ml) ethyl alcohol (70%) in 353 cases with the cure rates of 96% after the first injection and 98% after the second injection. All the abovementioned authors, except for Wyllie, had injected the agents submucosally and pararectally. However, we injected the agent only submucosally. Our success rates after the single injection was 96% and after the second injection was 98%.

We used 4.5 ml (1.5 ml in each site) in children less than 3 years and 6 ml in older children. In the abovementioned reports, the duration of rectal prolapse was not defined. Three patients in our report (2%) required two injections and three patients did not respond to the second injection.

These patients were more than 13 years of age. Each of these patients belonged to one of the above mentioned three groups and the duration was no longer than the responded patients. Although the clinical presentation of these patients was similar to others, probably the cause of prolapse was different from the other children. The cure rates in our patients were 96% after the first injection and 98% after the second injection, in spite of a prolonged duration. Quist *et al*.[1] stated that a duration of more than 8 weeks requires operation; however, all our patients responded to the sclerosing agent in spite of the prolonged rectal prolapse and no recurrence was detected after 1 year. No infectious complications were observed. Soilage in our patients was probably due to mucosal edema and not because of damage to the sphincter because we did not inject the sclerosing agent pararectally. Limping (observed in 4 patients) was probably due to the infiltration of the sclerosing agent outside the rectal wall at the level of the sciatic nerve.

We concluded that the submucosal injection of ethyl alcohol with smaller amounts (4-6 ml) is effective in prolonged rectal prolapse. It appears that the response to this treatment was not related to the site, amount of agent, type of agents and duration of disease. However, the secondary causes in older children despite of several anatomic variants in early childhood rectal prolapse are the main reasons for the failure of alcohol injection. All the patients with rectal prolapse beyond early ages should be evaluated for the secondary causes such as chronic constipation, neuromuscular disorders, scleroderma, Hirschsprung's disease, rectal polyp, cystic fibrosis and parasites.[7]

## CONCLUSION

We concluded that the children before adolescence do not require operative approach used for adults and they respond well to the sclerosing agents, particularly ethyl alcohol.
